# Filling the interspace—restoring arid land mosses: source populations, organic matter, and overwintering govern success

**DOI:** 10.1002/ece3.2448

**Published:** 2016-10-05

**Authors:** Lea A. Condon, David A. Pyke

**Affiliations:** ^1^ Department of Botany and Plant Pathology Oregon State University Corvallis OR USA; ^2^ U.S. Geological Survey Forest and Rangeland Ecosystem Science Center Corvallis OR USA

**Keywords:** biological soil crusts, *Bryum argenteum*, ecotypes, Great Basin, sagebrush ecosystem, shrub steppe, *Syntrichia ruralis*

## Abstract

Biological soil crusts contribute to ecosystem functions and occupy space that could be available to invasive annual grasses. Given disturbances in the semiarid shrub steppe communities, we embarked on a set of studies to investigate restoration potential of mosses in sagebrush steppe ecosystems. We examined establishment and growth of two moss species common to the Great Basin, USA:* Bryum argenteum* and *Syntrichia ruralis* from two environmental settings (warm dry vs. cool moist). Moss fragments were inoculated into a third warm dry setting, on bare soil in spring and fall, both with and without a jute net and with and without spring irrigation. Moss cover was monitored in spring seasons of three consecutive years. Both moss species increased in cover over the winter. When *Bryum* received spring irrigation that was out of sync with natural precipitation patterns, moss cover increased and then crashed, taking two seasons to recover. *Syntrichia* did not respond to the irrigation treatment. The addition of jute net increased moss cover under all conditions, except *Syntrichia* following fall inoculation, which required a second winter to increase in cover. The warm dry population of *Bryum* combined with jute achieved on average 60% cover compared to the cool moist population that achieved only 28% cover by the end of the study. Differences were less pronounced for *Syntrichia* where moss from the warm dry population with jute achieved on average 51% cover compared to the cool moist population that achieved 43% cover by the end of the study. Restoration of arid land mosses may quickly protect soils from erosion while occupying sites before invasive plants. We show that higher moss cover will be achieved quickly with the addition of organic matter and when moss fragments originate from sites with a climate that is similar to that of the restoration site.

## 1 Introduction

Worldwide, biological soil crusts (BSCs) are important ecosystem components. BSCs reduce eolian soil erosion (Belnap & Gillette, 1998), influence water infiltration (Chamizo, Cantón, Lázaro, Solé‐Benet, & Domingo, 2012), and nutrient cycling (Pendleton, Pendleton, Howard, & Warren, 2003), resulting in higher concentrations of essential nutrients in vascular plants grown in proximity to BSCs (Harper & Belnap, 2001). BSCs thrive in environments that are stressful to vascular plants (Bowker, Soliveres, & Maestre, 2010b) because they are poikilohydric, meaning that they tolerate dehydration and recover from it without physiological damage, making use of moisture as it becomes available (Proctor & Tuba, 2002). As stress tolerators, BSCs become more prominent along gradients of increasing aridity in Mexico (Aquilar, Huber‐Sannwald, Belnap, Smart, & Arredondo Moreno, 2009), Australia (Read, Duncan, Vesk, & Elith, 2011), Spain (Bowker et al., 2010b), China (Su, Li, Zheng, & Huang, 2009), and the southwestern United States (Bowker & Belnap, 2008). Although BSCs are common and important worldwide, they are rarely considered in restoration projects (Bowker, 2007). Given the high relative cover of BSCs in arid environments and their contribution to ecosystem processes (Belnap & Lange 2001), it is important to restore BSCs for their ecosystem functions. Of particular interest in the Great Basin, USA, is the ability of BSCs to reduce the establishment and time to germination of invasive annual *Bromus* species (Serpe, Orm, Barkes, & Rosentreter, 2006; Zaady, Boeken, Ariza, & Gutterman, 2003). Because ruderal *Bromus* species commonly invade disturbed environments in semiarid ecosystems of the western USA (Pyke, 2011), there is an increasing need to restore BSCs to reduce invasion by *B. tectorum* and other annual grasses.

Restoration of arid environments often incorporates techniques to facilitate native plant establishment by increasing soil moisture infiltration and availability (Bainbridge 2007). We tested techniques commonly used to increase soil moisture on the establishment and growth of arid land mosses, specifically the season of moss inoculation, the addition of organic matter, and irrigation. We decided to work with the moss component of BSCs because they are known to establish from gametophyte fragments alleviating any need to establish spores or provide additional mutual organisms (Serpe et al., 2006). Given that mosses are poikilohydric and have little ability to buffer changes in water availability (Wasley, Robinson, Lovelock, & Popp, 2006), it seems likely that all of these techniques aimed at increasing soil moisture could increase moss growth. Season of inoculation might influence the success of moss restoration because mosses need enough water for photosynthesis to outpace respiration leading to moss growth (Barker, Stark, Zimpfer, McLetchie, & Smith, 2005). Winter precipitation is the main moisture input in Mediterranean systems such as the Great Basin (Dobrowolski, Caldwell, & Richards, 1990). Additions of organic matter might favor mosses, as increased water holding capacity of litter leads to greater growth of bryophytes (Rincon, 1988). In the Great Basin, litter is often dominated by sagebrush leaves as domestic livestock often consume grasses (West, Provenza, Johnson, & Owens, 1984). Mosses are more prevalent under shrubs, as shrubs protect mosses from trampling (West, 1990) and their litter plus shade provide higher soil moisture (Bowker, Stark, McLetchie, & Mishler, 2000). The combination of organic matter and irrigation might ensure that mosses receive adequate moisture and that it is available longer to growing individuals when compared with either treatment alone.

When restoring vascular plant species, it is often important to select site‐appropriate ecotypes but we do not know whether the same is true for bryophytes. The distribution of a single vascular plant species over a range in climate is known to enhance development of ecotypes (Clausen, Keck, & Hiesey, 1948), which often vary in their ability to thrive under environmental conditions. The climatic gradient that exists over the Great Basin is substantial in regard to precipitation, ranging from 200 to 460 millimeters over sagebrush steppe sites surveyed by Knutson et al. (2014) (30‐year average, PRISM 2010). We anticipated that this range was large enough to lead to the development of ecotypes in mosses if they exist. In addition, widespread species may develop site‐specific mutations that lead to regionally different populations in respect to their growth requirements. Plant zones are currently being developed for vascular plants in the Great Basin to enhance restoration success of vascular plants (Bower, St. Clair, & Erickson, 2014), but to our knowledge, no one has examined the potential of ecotypes of common BSC mosses.

Our study is the first to date to examine the potential existence of moss ecotypes based on collections from widely different environmental settings and the restoration capability of common arid land mosses by utilizing factors thought to increase soil moisture. In a set of separate experiments, a moisture treatment experiment, a seasonal inoculation experiment, and an overwintering experiment, we pose the following questions regarding the restoration of two populations per species (one from a warm dry and one from a cool moist environment) of mosses common to the Great Basin, *Bryum argenteum* and *Syntrichia ruralis* grown in a third common garden site:

1. Do moisture treatments of jute net (organic matter) application, spring irrigation, or the interaction of the two increase cover of these mosses?
2. Does season of inoculation affect moss cover? Is there an interaction between jute net and season of inoculation on resulting moss cover?
3. Do mosses put on substantial growth over the winter?

## 2 Materials and methods

### 2.1 Study species and site descriptions

The study was conducted using two moss species: *Bryum argenteum* Hedw. and *Syntrichia ruralis* (Hedw.) F. Weber and D. Mohr. *Bryum argenteum* is a cosmopolitan and ruderal species (De Las Heras, Herranz, & Martinez, 1993; Esposito, Mazzoleni, & Strumia, 1999; Pisa, Werner, Vanderpoorten, Magdy, & Ros, 2013). *Syntrichia ruralis* is a later successional species (Esposito et al., 1999). *Bryum argenteum* gametophyte shoots have appressed, overlapping leaves and are generally short, between 0.5 and 1 mm tall, and cylindrical. *Syntrichia ruralis* gametophyte shoots are 4‐ to 20‐fold larger than *B. argenteum,* between 2 and 20 mm, with leaves spreading when hydrated and twisted around stems when dry. Both species were collected from two environmentally distinct locations to ascertain any measured effect of potential ecotypes on rate of establishment and growth in a third location with environmental conditions that produce a similar vascular plant community, but has a climate slightly between that of the two collection sites for the mosses. This scenario is common in the restoration of native plants, where seeds are collected from distant sites but ecotypes are matched to restoration sites based on climatic conditions (BLM 2015). Although a thorough investigation of ecotypes would have replicated by location and may have produced reciprocal gardens, we elected to collect soil from each site with their corresponding moss and restored the moss on its soil in the new common garden location to only address the climate aspect of the environment as an initial examination of the potential for ecotypes to exist.

The two collection sites were separated by 235 km: The Morley Nelson Snake River Birds of Prey National Conservation Area (BoP) south of Boise, Idaho, and the Steens Mountain Cooperative Management and Protection Area (Steens) south of Burns, Oregon, and were both managed by the Bureau of Land Management (Table 1). BoP is classified as having a mesic soil temperature and aridic soil moisture regime. In contrast, Steens has a frigid soil temperature and xeric soil moisture regime. The recipient common garden site, where the experiment was located, is on private land about 1.6 km north of Madras, Oregon, USA (Table 1). The recipient site, similar to BoP, is classified as having a mesic soil temperature and aridic soil moisture regimes (Chambers et al., 2014). During the 2013–2014 water year, this site received 178 mm of precipitation (89 mm between 1 October and 1 March), and in the 2014–2015 water year, it received 185 mm of precipitation (122 mm between 1 October and 1 March). Thirty‐year average precipitation at the site is 278.7 mm (PRISM 2010). Over the period of study, maximum temperature was 38°C on 27, 28 June and 3 July 2014 and minimum was −31°C on 9 December 2013. At the time of field application, maximum temperature was 23.6°C on 5 May 2013 and 8.94°C on 16 November 2013 (WRCC 2014). Mean humidity on those days was 41% and 63%, respectively.

**Table 1:** Environmental characteristics of moss collection sites and the recipient site

	Recipient (Madras, Oregon)44°43′36.54″N, 121°04′00.10″W	Birds of Prey, Idaho43°10′53.86″N, 116°03′43.76″W	Steens, Oregon 42°47′18.10″N, 118°39′46.40″W
Elevation	836 m	931 m	1430 m
Topographic Setting	Lava plains	Lava plateaus	Intermontane plateaus
Average Freeze‐free Period (USDA 2006)	125 days	127 days	60 days
30‐year Average Maximum Summer Temperature (PRISM 2010)	28.5°C	30.7°C	28.7°C
30‐year Average Minimum Winter Temperature (PRISM 2010)	−2.4°C	−3.9°C	−5.4°C
30‐year Average annual precipitation (PRISM 2010)	278.7 mm	251.0 mm	338.4 mm
Soils (Soil Survey Staff 2015)	Mesic, Aridic Haploxerolls	Mesic, Durinodic Haplocalcids and shallow, Typic Argidurids	Frigid, Lithic Argixerolls and frigid, Lithic Xeric Haplargids
Ecological Site Type (Soil Survey Staff, 2015)	Loamy 8‐10 PZ (203–254 mm), R023XY216OR	Calcareous Loam 7‐10 PZ (178–254 mm), R011XY010ID	Clay Pan 12‐16 PZ (305–406 mm), R023XY216OR
Dominant Vegetation for Ecological Site Type (Soil Survey Staff, 2015)	*Artemisia tridentata* spp. *wyomingensis* (Condon per obs.)Not listed	R011XY010ID: *Atriplex confertifolia*,* Picrothamnus desertorum,* and *Artemisia tridentata* spp. *wyomingensis*	R023XY216OR: *Artemisia arbuscula*, annuals, and *Poa secunda*

The location of each site is reported with latitude and longitude coordinates subtending their respective sites. PZ, precipitation zone.

In March and April of 2013, mosses and soil were collected from each site. Mosses were dried to ambient humidity within a laboratory at the US Geological, Forest and Rangeland Ecosystem Science Center in Corvallis, Oregon, USA. Moss fragments selected for use were apical leaves that remained green at the time of desiccation because green fragments recolonize more rapidly than older moss (Barker et al., 2005). Mosses were rubbed through a 2‐mm sieve to create fragments for even application of inoculants, stored, and transported in paper bags.

Soils were collected under the moss, no deeper than 10 cm, and used in the experiment because arid land mosses might show preferences for soil types (Bowker & Belnap, 2008). Using the procedure outlined in Thein (1979), surface soils from both BoP and Steens were determined to have a loamy texture. We did not test for any other differences between soils at the two sites, but field‐verified that the soils matched the soil map unit descriptions for soils mapped to those locations on the Web Soil Survey (http://websoilsurvey.sc.egov.usda.gov/ Accessed June 22, 2016). Soils were autoclaved to eliminate moss propagules and kept sterile until use. Seedling flats (12.7 cm × 17.8 cm × 5.1 cm deep) were initially filled halfway with soil from recipient site, and the remaining 2.54 cm was filled with soil from the collection location of the moss to be restored.

### 2.2 Experimental design

Both moss species were collected at BoP and Steens. We refer to each species in association with its collection location (species location, hereafter source population). We address each research question posed above as a separate experiment: moisture treatment experiment, seasonal inoculation experiment, and overwintering experiment, using moss from all source populations in each experiment (*Bryum*‐ST, *Bryum*‐BoP, *Syntrichia*‐ST, and *Syntrichia*‐BoP). The moisture treatment experiment tests the application of organic matter in the form of jute net (yes or no) and irrigation in the spring season only (yes or no) on the average cover of these mosses (Fig. S1). The seasonal inoculation experiment tests the season of inoculation (spring, 5 May 2013 and fall, 16 November 2013) and organic matter in the form of jute net (yes or no) on the average cover of mosses. The overwintering experiment included a subset of data from the moisture treatment experiment, using data from three sampling dates in total, a May date in 2013, 2014, and 2015 to assess whether mosses put on a significant amount of growth over the winter.

Moss fragments (1 g) were hydrated with approximately 60 ml of water for 20 min in the field immediately prior to being spread over the surface of a flat to yield an application rate equivalent to 43.5 g of moss per m^2^. If moss fragments receive enough water to begin photosynthesis but do not fix enough carbon to make up for what was expended in respiration, they become stressed and lose vitality (Stark, Brinda, & McLetchie, 2011). By hydrating mosses for 20 min prior to application, we ensured that moss fragments were actively photosynthesizing at the time of application (Proctor & Smirnoff, 2000), likely resulting in a net positive carbon balance and minimizing stress to individual fragments. Jute net was applied over mosses to increase water retention on the soil surface and the boundary layer experienced by the moss. Jute is plant fiber that is woven into nets for use in erosion control (Bainbridge 2007). Mosses were irrigated in the spring only because we anticipated the site would not be reliably accessible in the fall and winter due to muddy roads with high clay content. Irrigation consisted of watering flats with tap water to field capacity weekly during the first spring (May–June), after which ambient precipitation was the only source of water. Season of moss inoculation was tested with a spring (5 May 2013) and a fall (16 November 2013) application. Moss material was pooled by location, so we did not have replication of each location and could not statistically test for the effect of collection location on average moss cover. We scored ocular estimates of moss cover to the nearest 1% with a 12.7 cm × 17.8 cm gridded frame with 25.4 mm × 25.4 mm grids.

The experiment was conducted between April of 2013 and May of 2015. Replicates were flats and buried so the top of flat was flush with the native soil surface. Treatment combinations are shown in Fig. S1. Some flats were lost to disturbance, but no treatment had fewer than 11 flats. Flats were randomly located within four 1.8 m × 1.8 m × 0.6 m cages (width × length × height) to discourage wildlife damage. Cages were made from a frame of welded aluminum 2.5 cm × 2.5 cm square stakes, 0.6 m in height, with the sides draped in green plastic fencing attached with zip ties (1.9 cm × 1.9 cm mesh). Hog fence (7.6 cm × 20.3 cm) was welded across the top of the frame, enclosing the top of the cage. Hardware cloth (0.6 cm × 0.6 cm) was used along the base of each cage and to a height of 10.2 cm to exclude small mammals. This minimized the use of galvanized metal, which contains zinc, a common ingredient in moss control herbicides. Flats were arranged adjacent to one another and with roughly 5 cm between the outer flats and the sides of the cage. A section between flats was left open in the center of the block for accessibility when measuring. Our scope of inference is limited to the four previously mentioned source populations grown outdoors in Madras, Oregon, USA, between April 2013 and May 2015.

### 2.3 Data analysis

Data were analyzed with linear mixed models to allow for an unbalanced design and repeated measures of each flat fit by maximum likelihood. This approach does not allow for the calculation of traditional ANOVA tables. *F*‐values are used to report significance for variables of interest. Analyses were performed in R version 3.1.2. R Studio, Version 0.98.1091 was also used (R Core Team 2013). Research questions were addressed as three separate experiments: the moisture treatment experiment, the seasonal inoculation experiment, and the overwintering experiment with separate additive models being fit for each. We conducted our analyses as three separate experiments, with three separate models. This allowed us to address our research questions in a straightforward manner, and models were evaluated to ensure that they met assumptions of normality and symmetry. We fit full models, including three‐ and four‐way interactions where interactions were significant, because we were interested in quantifiable additive differences in percent moss cover given treatments and significant interactions among treatments (Fig. S1). For example, we wanted to quantify expected increases in mean percent cover of inoculating moss with jute net compared to inoculating moss without treatment. This translates into a difference in mean percent cover between treatments. In other words, we wanted to know how much of a benefit or detriment our treatments were in comparison with doing nothing more than inoculating a site with moss fragments in the spring. All lower order factors included in each significant interaction were kept in final models. The moisture treatment experiment model assessed the mean effect of each treatment combination on average moss cover, evaluating all main effects and interactions of four levels of source population, two levels of spring irrigation, and two levels of jute net. This model only included data from mosses inoculated in the spring of 2013. Using the difference in mean effects on cover, each treatment combination was compared to moss inoculated without additional treatments but from the same source population. The seasonal inoculation experiment model tested the effect of season of inoculation and the presence or absence of jute net on average moss cover using data from both seasons of inoculation between 7 April 2014 and 3 May 2015. Mean effects on cover of moss inoculated in the fall were subtracted from moss inoculated in spring both with and without jute net. The overwintering experiment model only included data from mosses inoculated in the spring of 2013 and evaluated the mean effect of overwintering once and twice on average moss cover for each treatment combination (Table S1), using data from one May sampling date in each of 3 years (26 May 2013, 25 May 2014, and 3 May 2015). Differences in mean effects of earlier years were subtracted from those of later years.

A random effect of flat identified by replicate number for each source population was used as flats were measured repeatedly through time. Flats were nested by cage (blocked by cage). Residuals met assumptions of normality and symmetry within years, so models were weighted by year. This was performed using a compound symmetry correlation structure to allow for heterogeneity of variance by year. Average cover of moss was the response variable. Confidence intervals were Bonferonni‐adjusted for multiple comparisons.

## 3 Results

Interactions in all three models were statistically significant, indicating that the observed change in moss cover, due to any one of the included factors, varied with the value of the other factors. In the moisture treatment experiment, *Bryum* from the BoP source population decreased in cover when receiving both irrigation in the spring and jute net, but increased with jute alone, necessitating a three‐way interaction in the moisture treatment experiment model between source population, jute net, and irrigation (*F*_3,184_ = 5.3, *p *= .002). Results of the seasonal inoculation experiment model also varied with source population resulting in higher cover of *Bryum* when inoculated under the jute treatment in the spring but having the opposite effects on cover of *Syntrichia* unless inoculated in the fall without jute, signifying a three‐way interaction in the seasonal inoculation experiment between source population, jute net, and season of inoculation (*F*_3,185_ = 3.2, *p *=* *.02). Moss cover from all source populations significantly increased in cover over each winter season with the addition of jute or the combination of jute and spring irrigation. Increases varied based on the combination of source population, irrigation, jute net, and year, indicating a four‐way interaction in the overwintering experiment (*F*_6,343_ = 4.7, *p *=* *.0001).

### 3.1 Moisture treatment experiment: source population, jute net, and spring irrigation

**Figure 1:**
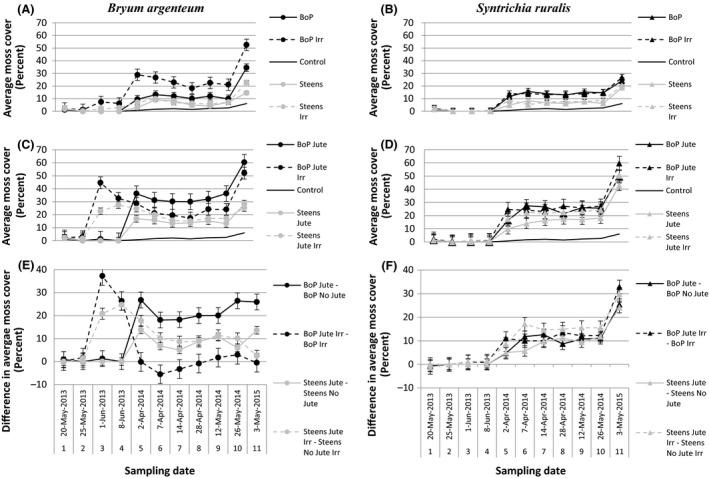
Average moss cover (percent) from treatment experiment on spring inoculations, by sampling date, over the course of the study. Graphs in the first column (A, C, and E) are of *Bryum argenteum,* and graphs in the second column (B, D, and F) are of *Syntrichia ruralis*. Graphs in the first row (A, B) show the effect of spring inoculation with and without irrigation. Graphs in the second row (C, D) show the effect of jute net with and without irrigation. Graphs in the third row (E, F) show a difference in moss cover with and without jute net between otherwise similar treatment. Abbreviations are as follows: BoP‐Birds of Prey, Idaho population, Steens, Oregon population, Jute‐jute net, and Irr‐irrigation. Weeks 1 and 5 did not include all possible replicates, but cover is averaged among all flats surveyed in that week. Weeks 1 and 5 were not used in the analysis because they were not complete datasets. Week 2 was not used because of a large number of zeros which broke assumptions of normality and symmetrical variance. Please note that there are gaps in our sampling between weeks 4 and 5 and weeks 10 and 11

**Figure 2:**
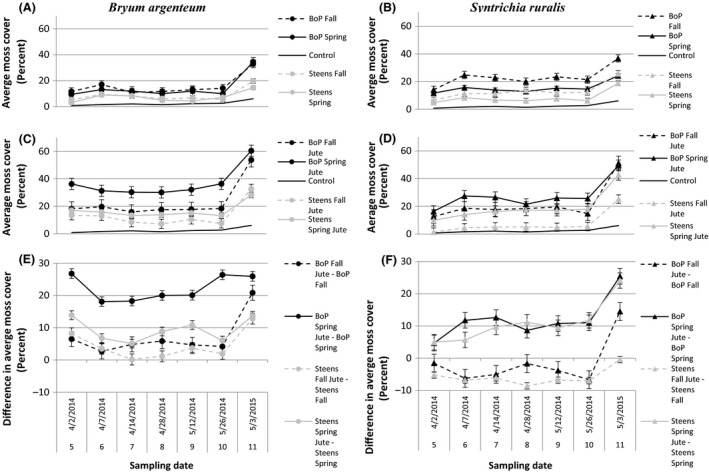
Average moss cover (percent) from season experiment on spring and fall inoculations by sampling date, over the course of the study. Graphs in the first column (A, C, and E) are of *Bryum argenteum,* and graphs in the second column (B, D, and F) are of *Syntrichia ruralis*. Graphs in the first row (A, B) show moss cover from both seasons of inoculation. Graphs in the second row (C, D) show moss cover with jute net treatment from both seasons of inoculation. Graphs in the third row (E, F) show a difference in moss cover with and without jute net between otherwise similar treatments. Abbreviations follow Fig. 1. Week 5 was not used in analysis because it did not include all possible replicates, but cover shown above is averaged among all flats surveyed in that week. Please note that there are gaps in our sampling between weeks 4 and 5 and weeks 10 and 11

Source populations reached different amounts of cover when inoculated in the spring (Fig. 1). *Bryum*‐BoP achieved twice as much cover as *Bryum*‐ST (34.5% vs. 14.6% cover without irrigation and 52.8% vs. 22.8% with irrigation) (Fig. 1). The same trend in source population was seen with *Syntrichia*‐BoP compared to *Syntrichia*‐ST (24.2% vs. 18.8% cover without irrigation and 26.8% vs. 20.4% with irrigation). The highest amounts of moss cover were observed for *Bryum*‐BoP when grown with jute net and for *Syntrichia*‐BoP when grown with the combination of jute net and irrigation (60.4% and 59.6%, respectively, Fig. 1). Irrigation initially had a significant positive effect on cover of *Bryum*‐BoP regardless of the presence of the jute net. However, when *Bryum*‐BoP and *Bryum*‐ST received jute net and irrigation concurrently, the combination resulted in a dieback of *Bryum*‐BoP and *Bryum*‐ST in the first year that recovered in later years (Fig. 1A,C). Irrigation did not result in increased growth or dieback of *Syntrichia* (Figs 1B,D, and 2).

**Figure 3:**
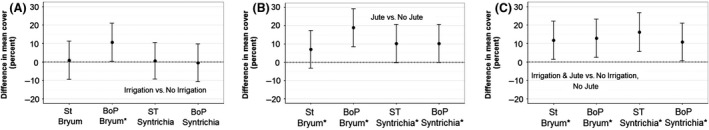
Differences in mean cover between mosses inoculated in the spring with a given treatment minus cover of those without treatments. Treatments considered include (A) irrigation, (B) jute net, and (C) the interaction between the two. Error bars represent 99% CI. *p *<* *.05 is indicated by an asterisk next to the source population

Jute net additions increased moss cover for all source populations as demonstrated by values above the zero line (Figs 1 and 3). The combination of jute net and irrigation also resulted in increases in mean moss cover for all source populations (Figs 1 and 3). By the end of the experiment, this was apparent for *Syntrichia*‐BoP and *Syntrichia*‐ST (Fig. 1F) but less so for *Bryum*‐BoP and *Bryum*‐ST that had to recover from diebacks, which reduced average cover (Fig. 1C,E).

### 3.2 Seasonal inoculation experiment: source population, season of inoculation, and jute net

**Figure 4:**
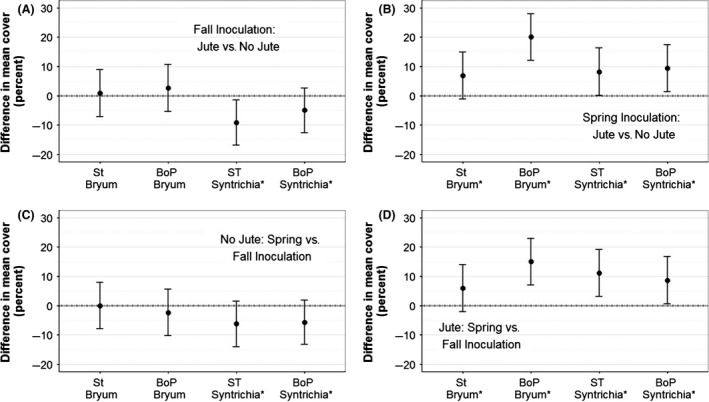
Differences in mean cover between mosses inoculated in the spring minus those inoculated in the fall, with and without jute. Error bars represent 99% CI. *p *<* *.05 is indicated by an asterisk next to the source population

Following both seasons of inoculation, *Bryum*‐BoP achieved greater cover than *Bryum*‐ST and *Syntrichia*‐BoP achieved greater cover than *Syntrichia*‐ST (Fig. 2). Resulting cover of mosses varied with season of inoculation. A fall inoculation resulted in a visible, but not statistically significant increase in cover of *Syntrichia*‐ST and *Syntrichia*‐BoP over a spring inoculation, although the same was not true for *Bryum*‐BoP or *Bryum*‐ST (Figs 2 and 4). *Syntrichia*‐BoP and *Syntrichia*‐ST achieved greater cover without jute net following a fall inoculation but achieved greater cover with jute net following a spring inoculation, with the BoP source population achieving greater cover (Fig. 2). All spring inoculations showed increased cover with jute as demonstrated by values above the zero line (Figs 2 and 4). By the end of the experiment, cover values of *Bryum*‐BoP, *Bryum*‐ST, and *Syntrichia*‐BoP with jute net started to converge regardless of season of inoculation, making season of inoculation a short‐term effect.

### 3.3 Overwintering experiment

Overwintering generally had a positive effect on moss cover that was visually more dramatic than any treatment effect (Fig. 1). This was apparent when looking at moss cover between the sampling dates of 8 June 2013 and 2 April 2014 and then again between 26 May 2014 and 3 May 2015. Moss growth in the winter months (overwintering) is represented by the difference in mean moss cover among years. Most single‐year comparisons and all differences between 2013 and 2015 were significant at a *p *<* *.05, with the exception of *Bryum*‐ST without treatment (Table S1). Winter moss growth resulted in long‐term positive effects on moss cover for all source populations. This analysis also corroborates the long‐term positive effect of jute net on moss cover seen in the moisture treatment experiment (Table S1).

## 4 Discussion

Although others have grown arid land mosses in greenhouses (Doherty, Antoninka, Bowker, Ayuso, & Johnson, 2015; Serpe et al., 2006), we are the first to rapidly grow arid land mosses in the field and specifically in the Great Basin Floristic Province (Lentz, 2000). The application of jute net resulted in increased cover of all source populations tested when compared with mosses that were inoculated without jute net. *Bryum*‐BoP achieved 25% more cover and *Bryum*‐ST achieved 15% more cover in the year following inoculation. *Syntrichia* from both locations achieved between 5% and 15% more cover in the first year after inoculation and 25%–35% more cover 2 years after inoculation. Irrigation had short‐term effects on moss cover, but long‐term positive increases resulted from the application of jute net and allowing growth over the winter. In the first 2 years of the study, the more ruderal species, *Bryum,* achieved higher cover over the later successional species, *Syntrichia*. By the third spring, both species with treatments achieved approximately 60% cover (Fig. 1).

Populations of both species collected from the warm dry location, BoP, reached higher cover than populations from the cool moist location, Steens. The superior performance of mosses from BoP and the similarity in climatic conditions between BoP and Madras suggest that mosses used for restoration should be gathered from sites that are climatically similar to restoration sites, as is practiced for plant communities (Bower et al., 2014; Knapp & Dyer, 1998). We did not replicate collection sites or common gardens nor did we conduct reciprocal inoculations, so the potential for ecotypes is indicated, but should be further evaluated with a broader set of sites and more detailed designs. Although edaphic properties might influence the restoration potential of mosses, we intentionally controlled for those here. The disturbance history of each site may also have selected for more vigorous individuals. The BoP collection sites appeared to have incurred more disturbance than the Steens collection sites given the predominance of ruderal species (Condon per obs.). These differences might represent a “physiological history” that is sometimes present in mosses and may take a “deacclimation” period to remove (Stark, Greenwood, Brinda, & Oliver, 2013).

Addition of jute net had long‐term effects of increasing cover of all source populations. It is likely that organic matter enhanced the boundary layer (Kimmerer, 2003) and therefore the length of available moisture experienced by the moss, although these effects cannot be separated from possibility that the jute net also held the moss in place, possibly decreasing soil surface temperature and exposure (Graf & Rochefort, 2010). The presence of jute net might mimic the soil properties present in intermediate successional stages of mosses, specifically humus‐rich soils (Esposito et al., 1999). Long‐term effects of applying jute net or other organic materials on all source populations tested (Fig. 1, Table S1) warrant its inclusion with the reintroduction of mosses.

A potential application of mosses in the Great Basin is during postwildfire rehabilitation. Postfire rehabilitation sometimes includes spreading rice straw as a hillslope protection against soil erosion (Robichaud, Ashmun, & Sims, 2010). Ours results indicate the potential for including arid land mosses during the distribution of straw, where straw may both retain the moss and provide a boundary layer, while the moss establishes and protects the soil surface, during the initial growing season, when vascular plants are recovering from fire.

Season of inoculation had short‐term positive effects on moss cover when mosses were inoculated in spring with jut net (Figs 1, 2, and 4). Regardless of the season of inoculation, mosses from all source populations significantly increased in cover during winter months (Fig. 1), making mosses ideal candidates for restoration efforts in either season. This is not surprising given that mosses are both immune to most freezing events, as the formation of ice causes mosses to desiccate before freezing (Malek & Bewley, 1978), and they are poikilohydric, able to opportunistically use water as it is available as dew or precipitation provided mosses are capable of maintaining a positive carbon gain.

Effects of irrigation on moss cover were mixed and short‐term. Our efforts to irrigate mosses in the spring had little to no effect on *Syntrichia* and led to spikes in cover of *Bryum* followed by diebacks, necessitating two seasons for mosses to recover to predieback levels. These findings corroborate Reed et al. (2012) who observed diebacks in mosses following increases in small summer precipitation events. Rapid drying events for mosses result in negative net carbon gain (Alpert, 2000) through the breakdown of thylakoid membranes and chlorophyll a (Schonbeck & Bewley, 1981). A single rapid drying event can result in 60% tissue death and 90% following two successive events (Stark et al., 2011). Mosses that survive rapid drying events demonstrate reduced vitality, net photosynthesis, and de‐greening of tissues (Schonbeck & Bewley, 1981). For a moss in the Great Basin, spring irrigation may result in a rapid drying event. On May 5 at the experiment site, the spring inoculation date, the temperature was warm at 23.6°C and the low relative humidity was 17%, reaching 30.6°C with a low relative humidity of 23% on June 7th. Irrigation treatments followed May 5 and continued until June 8. Our study shows that irrigation, under warm conditions, should be avoided in moss restoration efforts.

Following disturbance in the Great Basin, sites with higher perennial herbaceous cover demonstrate increased site resistance, the ability to inhibit invasion by *B. tectorum* (Condon, Weisberg, & Chambers, 2011). By restoring the moss component of BSCs, we are facilitating perennial herbaceous cover, as the presence of BSCs is associated with increased concentrations of essential nutrients (Harper & Belnap, 2001) and higher seed production (DeFalco, Detling, Tracy, & Warren, 2001) in vascular plants. Mosses facilitate establishment of perennial grasses (St. Clair, Webb, Johansen, & Nebeker, 1984) and *Artemisia* species (Su et al., 2009). Other ecosystem functions provided by mosses include buffered soil temperatures (Gornall, Woodin, Jonsdottir, & Van der Wal, 2009), which might explain why mosses facilitate the germination of some vascular plants (Su et al., 2009) and increase seedling survivorship (St. Clair et al., 1984).

Worldwide, mosses are present in the earliest stages of primary succession as demonstrated by the presence of moss on the tephra surface deposited by the eruption of Mount Saint Helens (Zobel & Antos, 1997). Mosses can grow rapidly on unstable (Esposito et al., 1999) or sandy (Bowker & Belnap, 2008) substrates, increasing soil stability and protecting against raindrop splash erosion (Williams, Dobrowolski, & West, 1995a). This indicates that mosses may be an appropriate restoration material in heavily disturbed areas, especially given that moss establishment is a positive feedback on newly exposed terrain (Bowden, 1991), influencing soil properties (De Las Heras et al., 1993) and adding carbon and nitrogen as mosses are often associated with cyanobacteria (Arróniz‐Crespo et al., 2014).

Our study demonstrates that high amounts of moss cover can be achieved with the use of jute net and properly selected source populations, in a region where restoration efforts are often unsuccessful at restoring high amounts of vascular plant cover (Knutson et al., 2014). We recognize the limited spatial inference related to this study, but we believe these results warrant future work that expands on these findings. Developments leading to moss increases for commercial production have been demonstrated (Antoninka, Bowker, Reed, & Doherty, 2016) and may lead to wider moss restoration studies in the future. In addition to mosses, studies relating to restoration of additional biological soil crust species are needed to maintain ecosystem functions (Bowker, Maestre, & Escolar, 2010a). Use of slurries has also been shown to be effective for growing cyanobacteria (Maestre et al., 2006). The affinity of arid land mosses for wetter environments is shared with gelatinous, nitrogen‐fixing lichens such as *Collema* sp., (Davidson, Bowker, George, Phillips, & Belnap, 2002), suggesting that similar restoration treatments may also favor these lichens. The methods proposed here could be used to facilitate not only arid land mosses but also cyanobacteria and early successional lichens.

## Funding Information

National Landscape Conservation System, Research and Science Program, Bureau of Land Management, (Grant/Award Number: Interagency Agreement L12PG00159_00).

## Conflict of Interest

None declared.
