# Supplementary material for: Filling the interspace—restoring arid land mosses: source populations, organic matter, and overwintering govern success
Source: Ecol Evol. 2016 Oct 5;6(21):7623–32. doi: 10.1002/ece3.2448 (PMC6093148; doi:10.1002/ece3.2448)
Supplement: Supplementary file 1 [file ECE3-6-7623-s001.docx]

**Supporting Information**

**Additional Supporting Information may be found in the online version of this article:**

Table S1. Differences in mean (percent) cover (dCover) between years (overwintering periods) of mosses inoculated in the spring from a given treatment combination.

Figure S1. Schematics of A.) the treatment experiment (testing the effects of source population, spring irrigation and jute net on moss cover) and B.) the seasonal inoculation experiment (testing the effects of source population, season of inoculation and jute net on moss cover).

Table S1. Differences in mean (percent) cover (dCover) between years (overwintering periods) of mosses inoculated in the spring from a given treatment combination. * Indicates that there is a significant difference for this listed treatment and source population for the years compared at a *P* < 0.05 and a 99% CI that does not cross zero.

| **Treatment** | **Years Being Compared** | **dCover (99.9% CI)** | ***P***  **(df = 352)** | **dCover (99.9% CI)** | | ***P***  **(df = 352)** | **dCover**  **(99.9% CI)** | | ***P***  **(df = 352)** | | **dCover (99.9% CI)** | ***P***  **(df = 352)** | |
| --- | --- | --- | --- | --- | --- | --- | --- | --- | --- | --- | --- | --- | --- |
|  | | ***Bryum argenteum***  **from Birds of Prey** | | ***Bryum argenteum***  **from Steens** | | | ***Syntrichia ruralis***  **from Birds of Prey** | | | | ***Syntrichia ruralis***  **from Steens** | | |
| No Treatment | 2015 vs 2014  2014 vs 2013  2015 vs 2013 | 24.6*  (10.3 to 38.8)  9.9*  (1.8 to 18.1)  34.5*  (17.2 to 51.8) | <0.001  <0.001  <0.001 | 7.4  (-6.8 to 21.7)  7.2  (-1.0 to 15.3)  14.6  (-2.7 to 31.9) | 0.08  <0.004  <0.005 | | 9.5  (-4.1 to 23.2)  14.6*  (6.8 to 22.4)  24.2*  (7.5 to 40.8) | 0.02  <0.001  <0.001 | | 12.5  (-1.7 to 26.7)  6.3  (-1.9 to 14.2)  18.8*  (1.5 to 36.0) | | | 0.004  0.011  0.0004 |
| Irrigation | 2015 vs 2014  2014 vs 2013  2015 vs 2013 | 31.5*  (17.3 to 45.7)  19.5*  (11.4 to 27.7)  51.0*  (33.8 to 68.4) | <0.001  <0.001  <0.001 | 15.7*  (1.4 to 29.9)  6.8  (-1.3 to 15.0)  22.5*  (5.2 to 39.8) | <0.001  <0.006  <0.001 | | 11.9  (-2.3 to 26.2)  14.8*  (6.7 to 23.0)  26.8*  (9.5 to 44.0) | <0.006  <0.001  <0.001 | | 11.8  (-1.4 to 25.0)  8.6*  (1.1 to 16.2)  20.4*  (4.4 to 36.4) | | | 0.003  <0.001  <0.001 |
| Jute net | 2015 vs 2014  2014 vs 2013  2015 vs 2013 | 24.1*  (9.8 to 38.3)  36.3*  (28.2 to 44.5)  60.4*  (43.1 to 77.7) | <0.001  <0.001  <0.001 | 15.1*  (0.9 to 29.3)  13.2*  (5.0 to 21.3)  28.3*  (11.0 to 45.5) | <0.001  <0.001  <0.001 | | 23.9*  (9.0 to 38.8)  25.6*  (17.1 to 34.1)  49.5*  (31.5 to 67.6) | <0.001  <0.001  <0.001 | | 22.5*  (7.7 to 37.4)  18.0*  (9.5 to 26.5)  40.5*  (22.5 to 58.6) | | | <0.001  <0.001  <0.001 |
| Irrigation x Jute net | 2015 vs 2014  2014 vs 2013  2015 vs 2013 | 28.0*  (13.5 to 42.5)  20.8*  (12.2 to 29.3)  48.8*  (31.4 to 66.0) | <0.001  <0.001  <0.001 | 8.1  (-6.2 to 22.3)  15.2*  (7.0 to 23.3)  23.3*  (6.0 to 40.5) | 0.06  <0.001  <0.001 | | 32.4*  (18.2 to 46.7)  27.2*  (19.0 to 35.3)  59.6*  (42.3 to 76.9) | <0.001  <0.001  <0.001 | | 24.6*  (9.8 to 39.5)  24.2*  (15.7 to 32.7)  48.8*  (30.8 to 66.9) | | | <0.001  <0.001  <0.001 |

A.

| **Source Population** | **Spring Irrigation** | **Jute Application** |
| --- | --- | --- |
| Bryum-St | Irrigation | Jute |
|  |  | No Jute |
|  | No Irrigation | Jute |
|  |  | No Jute |
| Bryum-BoP | Irrigation | Jute |
|  |  | No Jute |
|  | No Irrigation | Jute |
|  |  | No Jute |
| Syntrichia-St | Irrigation | Jute |
|  |  | No Jute |
|  | No Irrigation | Jute |
|  |  | No Jute |
| Syntrichia-BoP | Irrigation | Jute |
|  |  | No Jute |
|  | No Irrigation | Jute |
|  |  | No Jute |

B.

| **Source Population** | **Season of Inoculation** | **Jute Application** |
| --- | --- | --- |
| Bryum-St | Spring | Jute |
|  |  | No Jute |
|  | Fall | Jute |
|  |  | No Jute |
| Bryum-BoP | Spring | Jute |
|  |  | No Jute |
|  | Fall | Jute |
|  |  | No Jute |
| Syntrichia-St | Spring | Jute |
|  |  | No Jute |
|  | Fall | Jute |
|  |  | No Jute |
| Syntrichia-BoP | Spring | Jute |
|  |  | No Jute |
|  | Fall | Jute |
|  |  | No Jute |

Figure S1. Schematics of A.) the treatment experiment (testing the effects of source population, spring irrigation and jute net on moss cover) and B.) the seasonal inoculation experiment (testing the effects of source population, season of inoculation and jute net on moss cover). The treatment experiment only includes mosses inoculated in the spring. Treatment combinations from the treatment experiment were compared between years (2015 vs 2014, 2015 vs 2013, and 2015 vs 2013) in the overwintering experiment.
